# Fluorescein-guided surgery in high-grade gliomas: focusing on the eloquent and deep-seated areas

**DOI:** 10.1007/s00432-024-05796-1

**Published:** 2024-05-25

**Authors:** Yao Xiao, Mingrui Li, Xiangyu Wang, Jun Tan, Chaoying Qin, Qing Liu

**Affiliations:** Department of Neurosurgery, Xiangya Hospital, Central South University, Changsha, China

**Keywords:** Sodium fluorescein-guided surgery, Fluorescence, HGGs, Eloquent tumors, Deep-seated tumors, Extent of resection

## Abstract

**Purpose:**

The vital function of eloquent and deep brain areas necessitates precise treatment for tumors located in these regions. Fluorescein-guided surgery (FGS) has been widely used for high-grade gliomas (HGGs) resection. Nevertheless, the safety and efficacy of utilizing this technique for resecting brain tumors located in eloquent and deep-seated areas remain uncertain. This study aims to assess the safety and extent of resection of HGGs in these challenging tumors with fluorescein and explore its impact on patient survival.

**Methods:**

A retrospective analysis was conducted on the clinical and radiological data of 67 consecutive patients with eloquent or deep-seated HGGs who underwent surgery between January 2020 and June 2023. Lacroix functional location grade was used to determine the eloquence of the tumors. The comparison between the fluorescence-guided surgery group (FGS, *n* = 32) and the conventional white-light microscopic surgery group (non-FGS, *n* = 35) included assessments of extent of resection (EOR), rates of gross total resection (GTR, 100%) and near-total resection (NTR, 99 to 98%), postoperative Neurologic Assessment in Neuro-Oncology (NANO) scores, overall survival (OS), and progression-free survival (PFS), to evaluate the safety and efficacy of fluorescein-guided technology in tumor resection at these specific locations.

**Results:**

Baseline of demographics, lesion location, and pathology showed no significant difference between the two groups. GTR of the FGS group was higher than the non-FGS group (84.4% vs. 60.0%, OR 3.60, 95% CI 1.18–10.28, *p* < 0.05). The FGS group also showed higher GTR + NTR (EOR ≥ 98%) than the non-FGS group (93.8% vs. 65.7%, OR 7.83, 95% CI 1.86–36.85, *p* < 0.01). 87.0% of eloquent tumors (Lacroix grade III) in the FGS group achieved GTR + NTR, compared to 52.2% of control group (OR 6.11, 95% CI 1.50–22.78, *p* < 0.05). For deep-seated tumors, the rate of GTR + NTR in the two groups were 91.7% and 53.3%, respectively (OR 9.62, 95% CI 1.05–116.50, *p* < 0.05). No significant difference of the preoperative NANO score of the two groups was found. The postoperative NANO score of the FGS group was significantly lower than the non-FGS group (2.56 ± 1.29 vs. 3.43 ± 1.63, *p* < 0.05). Median OS of the FGS group was 4.2 months longer than the non-FGS group despite no statistical difference (18.2 months vs. 14.0 months, HR 0.63, 95% CI 0.36–1.11, *p* = 0.112), while PSF was found significantly longer in FGS patients than those of the non-FGS group (11.2 months vs. 7.7 months, HR 0.59, 95% CI 0.35–0.99, *p* < 0.05).

**Conclusion:**

Sodium fluorescein-guided surgery for high-grade gliomas in eloquent and deep-seated brain regions enables more extensive resection while preserving neurologic function and improve patient survival.

**Supplementary Information:**

The online version contains supplementary material available at 10.1007/s00432-024-05796-1.

## Introduction

Gliomas are the most common intracranial tumors, High-grade gliomas (HGGs) refer to WHO grade 3 and 4 tumors, representing the highest degree of malignancy. The survival rates at 1-, 3-, and 5-year for HGGs are 64.09%, 29.26%, and 15.26%, respectively (Qu et al. 2021), while the most aggressive type, GBM, has a median survival of only 16 months (Ho et al. 2014). The highly invasive nature of HGGs complicates the identification of tumor borders during surgery. Studies have confirmed that extent of resection (EOR) is the most significant prognostic indicator, making maximal safe resection as the mainstay of cytoreductive surgical strategy (Marko et al. 2014). Gross total resection (GTR), aiming to completely remove the contrast-enhancing (CE) area on MRI images, is most significantly associated with prolonged survival in HGGs (Han et al. 2020), which substantially improves the overall survival (OS) and progress free survival (PFS) of GBM patients compared to subtotal resection (STR) and biopsy only (Brown et al. 2016). To achieve a more thorough removal of infiltrating tumor cells, resection is extended to include non-contrast enhancing (NCE) part, a concept known as supramarginal resection, which has been recently introduced. Beyond GTR, supramarginal resection offered promising benefits to GBM patients, leading to a prolonged median OS ranging from 5 to 25 months and an extended PFS by up to 19 months (Wang et al. 2021). Nevertheless, an overly aggressive EOR may exacerbate patients’ neurologic deficits and even result in iatrogenic functional impairments. Unfortunately, over 50% of HGGs are located in eloquent regions of the brain (Gerritsen et al. 2022), potentially leading to language, motor, sensory, and cognitive deficits upon injury. Moreover, deep-seated tumors are challenging to expose, which impedes achieving the intended EOR, thereby compromising surgical outcomes. Surgery under conventional white-light microscope often struggles to accurately delineate tumor boundaries, prompting the development of various adjunct tools to maximize safe tumor resection, including neuronavigation, intraoperative MRI, intraoperative ultrasound, and fluorescence guidance.

Fluorescein is a fluorescent agent that readily binds to blood proteins upon administered intravenously, lacking tissue specificity. Similar to the mechanism of gadolinium, it infiltrates extracellular space at sites where the blood-brain barrier (BBB) is disrupted by HGGs. With excitation wavelengths between 460 and 500 nm and emission wavelengths from 540 to 690 nm, it enables tumor tissue to appear yellow-green under a microscope equipped with a YELLOW 560 filter (Wang et al. 2021). Sodium fluorescein is a commonly used tracer in clinical practice due to its affordability, rapid excretion, and minimal adverse reactions, leading to its increasing utilization by surgeons for intraoperative guidance. Emerging studies suggest that the intravenous administration of a low dose of 5 mg/kg sodium fluorescein after intubation achieves favorable intraoperative fluorescence in HGGs, thus increasing the EOR (Falco et al. 2019; Kutlay et al. 2021; Luzzi et al. 2021; Schebesch et al. 2022; Xi et al. 2023). Following a meta-analysis of 10 case-control studies, Smith et al. concluded that the use of fluorescence-guided surgery (FGS) can lead to a 29.5% increase in GTR rate of HGGs (Smith et al. 2021). However, assessment of fluorescence intensity during FGS in the FLUOCERTUM study, which included a cohort of 143 cases of diffuse astrocytomas and oligodendrogliomas, revealed that in 132 cases of HGG resection, fluorescence intensity was beneficial for tumor resection, whereas in all 11 cases of low-grade gliomas (LGGs), the fluorescence intensity was inadequate, showing only spotted fluorescence and offering limited assistance in tumor resection (Falco et al. 2019). Additionally, researchers have reported that the distribution of sodium fluorescein under microscope exceeded the CE area on T1-weighted MRI images (Neira et al. 2017; Bowden et al. 2018), implying that FGS might contribute to achieving gross-total resection, despite the risk of potential over-resection. Therefore, establishing whether the resection of tumors within the fluorescent border, particularly those in eloquent areas, can effectively achieve maximal safe resection is of importance. Another fact about fluorophores is that only when the tumor is sufficiently exposed can fluorescence be activated for thorough tumor resection. Thus, additional evidence is required to establish whether deep-seated tumors, which are challenging to expose, can benefit from FGS. However, there is a lack of research specifically evaluating FGS in eloquent and deep-seated HGGs.

The present study retrospectively compared the EOR and prognostic outcomes of patients with newly diagnosed eloquent or deep-seated HGGs who underwent FGS versus those who underwent conventional white-light surgery at our institution from January 2020 to June 2023. The main objectives of this study were to evaluate the extent of tumor resection, alterations in neurologic function, and postoperative outcomes following surgeries with or without fluorescence-guidance technique. The reporting of this study conforms to the STROBE statement (https://www.strobe-statement.org).

## Materials and methods

### Patients

Clinical data of newly diagnosed HGG patients who underwent surgical resection from January 2020 to June 2023 were collected and subjected to retrospective analysis. Informed consent was obtained before surgeries from the patients for the surgical procedure, intraoperative application of sodium fluorescein, and potential participation in medical research. This research protocol has been approved by the Ethics Committee of Xiangya Hospital, Central South University (No. 202403062) and all procedures adhered to the Helsinki Declaration.

Inclusion criteria included: (1) newly diagnosed HGG patients meeting the WHO CNS5 classification for Grade 3 and 4 gliomas; (2) eloquent tumors meeting Lacroix grade II or III according to the previous study (Lacroix et al. 2001) or deep-seated tumors originated in basal ganglia, insular, corpus callosum, thalamus, and brainstem etc.; (3) surgical resection of tumor via craniotomy, with or without intraoperative sodium fluorescein usage; (4) proper postoperative treatment was administered, including the standard Stupp protocol (Stupp et al. 2005) and alternative adjuvant therapy involving Temozolomide, CCNU, Bevacizumab, and Anlotinib etc.; (5) cases with complete demographic data, pre- and postoperative images, neurologic evaluation data, and follow-up information. Cases with histopathologically-confirmed LGGs, non-eloquent lesions, or those who underwent biopsy only were excluded.

### Clinical and radiological evaluation

Age, sex, perioperative laboratory test results and postoperative complication records of enrolled cases were obtained from the Hospital Information System. Cases receiving periodic MRI and adjuvant chemoradiotherapy were incorporated into survival analysis.

Pre- and postoperative neurologic and radiological assessments were conducted for subsequent analysis. We employed the Neurologic Assessment in Neuro-Oncology (NANO) scale to evaluate patients’ neurologic function, including 9 domains of gait, strength, ataxia, sensation, visual fields, facial strength, language, level of consciousness, and behavior (Nayak et al. 2017). The advantage of this system lies in its ability to provide a simple and rapid comprehensive evaluation of neurologic function. The NANO scale at admission and 3 months postoperatively was utilized to evaluate and compare the neurologic outcomes between the FGS cohort and the non-FGS cohort.

All enrolled cases included complete contrast-enhanced brain MRI scans taken within 7 days before the resection and 72 h postoperatively. Lesions involving the cortex and white matter fiber tracts also underwent diffusion tensor imaging (DTI) and blood oxygenation level-dependent (BOLD) functional MRI (fMRI) to assist in planning the resection. EOR is defined as the percentage of the difference between pre- and postoperative tumor volumes relative to the preoperative tumor volume. The stratification of EOR is based on the previous study: GTR, near-total resection (NTR), and STR were defined as 100%, 99 to 98%, and ≤ 97%, respectively (Luzzi et al. 2021). The tumor volume was calculated by manual segmentation on the T1-weighted MR images using an open-source software (Horos for Macintosh, version 3.3.6, https://www.horosproject.org) by a single blinded researcher.

### Fluorescence application and surgical protocol

Asleep craniotomy was performed in all cases. Following anesthesia induction in the FGS group, a small dose of 3–5 mg/kg of sodium fluorescein (Alcon Laboratories, Inc., USA) was intravenously administered. The surgical approach was planned based on preoperative radiological evaluation of tumor location and the proximity to functional areas and white matter fiber tracts. With the integration of a fluorescence filter YELLOW 560 (KINEVO 900, Carl Zeiss, Germany) into the surgical microscope, sodium fluorescein was effectively illuminated, producing a yellow-green fluorescence that aided in the operative procedure. After exposure of the lesion, resection was started with microscopic observation and inspection under white light. In the FGS group, the microscope was switched alternatively between white light and fluorescence, allowing for the simultaneous observation of anatomical structure and tumor boundaries in the surgical field. The surgery aimed for maximal safe resection, employing an inside-out approach to excise the tumor until reaching the non-fluorescent tumor margins. In contrast, the non-FGS group underwent resection based on the surgeon’s experiential judgment of tumor boundaries and intraoperative navigation information.

For tumors located within or adjacent to eloquent areas, we routinely used intraoperative neurophysiological monitoring (IONM) and brain mapping techniques, including motor evoked potentials (MEP), somatosensory evoked potentials (SSEP), direct cortical stimulation (DCS), direct subcortical stimulation (DsCS), brain-stem auditory evoked potentials (BAEP) and electromyography (EMG). The warning threshold was set as a 50% reduction in amplitude or a 10% extension in latency of compound muscle action potential (CMAP). In cases where tumors were located near language areas, multimodal techniques were employed. This involves integrating preoperative data of language cortices identified by BOLD-fMRI and reconstructed language tracts data into neuronavigation (Brainlab AG, Germany) to guide surgical planning. Furthermore, intraoperative neuronavigation and mapping/monitoring were used to ensure the maximal safe resection of these tumors. For deep-seated or small tumors that are challenging to localize, neuronavigation system was employed prior to resection to determine their precise location.

**Fig. 1:**
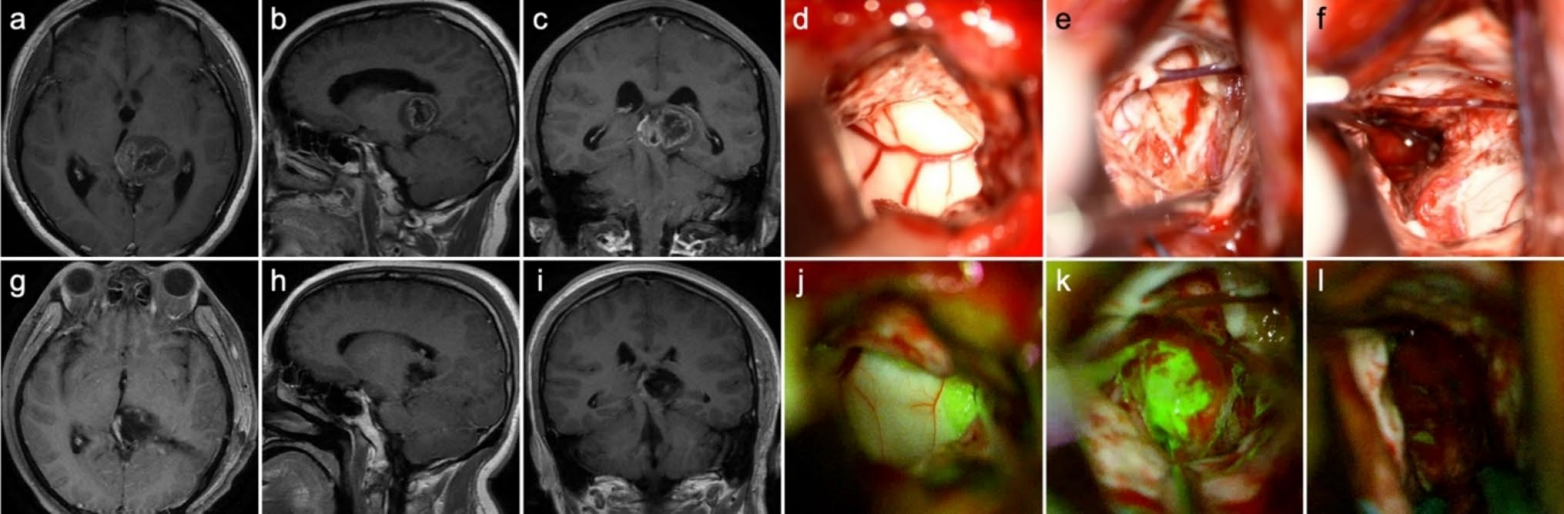
MR images and intraoperative fluorescence of a 38-year-old male with diffuse midline glioma, H3 K27-altered, CNS WHO grade 4. **a–c** Preoperative CE T1 WI images show a mass located in the left thalamus with cystic component, necrosis, and uneven enhancement within the tumor. **g**–**i** Postoperative CE T1 WI indicates a complete resection of the tumor. Through the transcortical-transtemporal approach, the deep-seated tumor was accessed. The surface of the thalamus was distended under white light (**d**) and showed no fluorescence under YELLOW 560 filter (**j**). **e** The tumor tissue with was detected upon incision of the thalamic cortex. **k** The tumor emitted a bright green signal when exposed to the light through YELLOW 560 filter. The tumor was removed completely (**f**), without fluorescence observed (**l**)

**Fig. 2:**
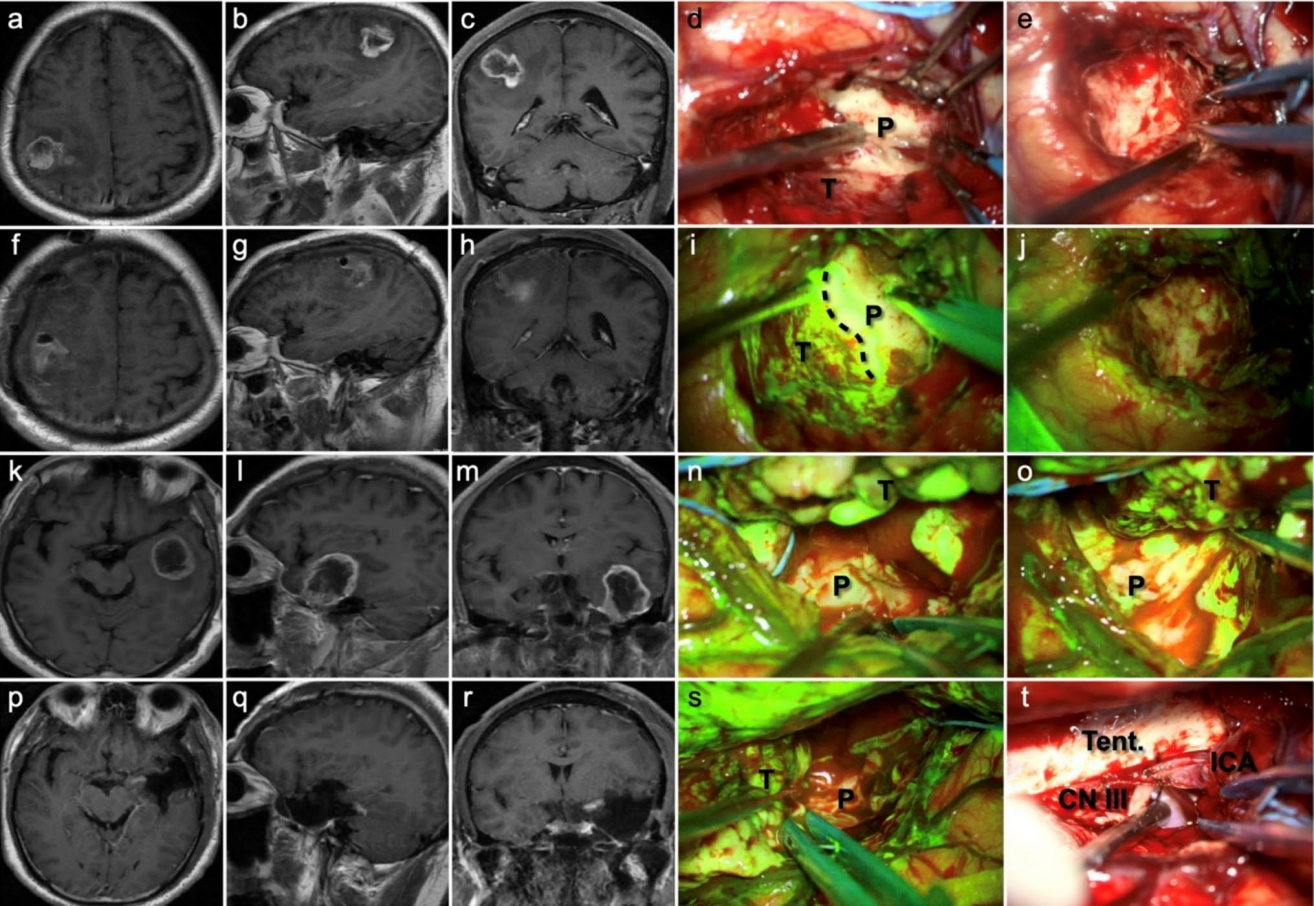
MR images and intraoperative fluorescence of a 63-year-old male (**a–j**) and a 59-year-old male (**k**–**t**), both with glioblastoma, CNS WHO grade 4. **a**–**c** Preoperative CE T1 WI images show a mass located in the right precentral gyrus with necrosis, and uneven enhancement within the tumor. Numbness in the right-hand fingers was noted by the patient before the tumor was detected. **f**–**h** Postoperative CE T1 WI images suggest a complete resection of the tumor. **d**, **i** Intraoperative photos of the tumor tissue and the adjacent brain parenchyma were exposed under white-light microscopy and fluorescent view, respectively (black dashed line presents the boundary line). The tumor tissue exhibits mosaic-like green fluorescence under YELLOW 560 filter. The tumor was completely excised (**e**) with no residual fluorescent signals detected (**j**). **k**–**m** Preoperative CE T1 WI images show the tumor was located in the left temporal lobe, close to the Wernicke’s area. The patient experienced memory loss and aphasia before the tumor was identified. **p**–**r** Complete resection of the tumor was achieved. **n**, **o**, **s** The tumor was excised by following the edematous zones along the tumor boundary under fluorescence guidance. **t** Complete resection of the tumor was performed, revealing the internal structures including the tentorial edge, oculomotor nerve (CN III), and internal carotid artery. *T* tumor, *P* parenchyma, *Tent.* tentorium, *ICA* internal carotid artery

Figure 1 exhibits the pre- and postoperative enhanced T1-weighted image, as well as intraoperative fluorescent feature, of a left-sided thalamic diffuse midline glioma. Through the transcortical-transtemporal approach, the deep-seated lesion was accessed at the trigone of left lateral ventricle. No obvious green fluorescence was observed on the surface of the thalamus; however, upon incision of the thalamic cortex, tumor tissue and highly intense of fluorescence were detected. The tumor was completely removed under the guidance of fluorescence. Figure 2 illustrates two cases of glioblastoma located in eloquent areas, one in the right precentral gyrus and the other in the left Wernicke’s area. Complete tumor resection was achieved along the peritumoral edematous zones using fluorescence guidance.

All tumor removals were carried out by a single, highly skilled neurosurgeon (Q.L.) who had been thoroughly trained in the application of sodium fluorescein, including cases both before the initiation of FGS at our institute (2020–2021) and the subsequent FGS (2021–2023).

### Evaluation of postoperative complications

Postoperative complications were graded according to a four-grade scale, which was proposed based on the therapy used to treat the complications: grade I, any non-life-threatening complications treated without invasive procedures; grade II, complications requiring invasive management; grade III, life-threatening complications; and grade IV, deaths as a result of complications.

### Endpoints and outcomes

The primary endpoint of this study was the difference in EOR between the FGS and non-FGS groups. Secondary endpoints included changes in the NANO scores for both groups, as well as assessments of survival outcomes. In accordance with the RANO 2.0 criteria, disease progression is defined as follows: ≥ 25% increase in the sum of (non)enhancing target lesions; unequivocal progression of existing (non)enhancing nontarget lesions; or new (non)enhancing lesions. OS and PFS are defined, respectively, as the duration of patient survival from the time of surgery until the last follow-up visit and the interval from surgery to the detection of tumor progression.

### Statistical analysis

RStudio (version 2023.06.1 + 524 for Macintosh) and Prism 9 (GraphPad Software, version 9.5.0 for Macintosh) were used for statistical analysis in the present study. Continuous variables were expressed as mean ± standard deviation, and categorical variables were expressed as frequencies or percentages. Comparisons for numerical variables between two groups were carried out using *t* test and Mann–Whitney test, and Fisher’s exact test were employed for categorical variables. Survival was estimated using Kaplan–Meier analysis, and comparison between the groups was assessed using the log-rank test. Statistical significance was set at *p* < 0.05.

## Results

Sixty-seven patients diagnosed with HGGs who underwent tumor resection were included in the analysis, with 32 undergoing FGS and 35 undergoing non-FGS. Patients ranged in age from 7 to 73 years, with an average age of 44 years. Thirty-four (50.7%) out of the included patients were female. There was no statistically significant difference in demographic data between the two groups (Table 1). All tumors presented as single lesions. The majority of lesions in both groups were located on the left side and within the eloquent areas (Lacroix functional location grade III). Twelve (37.5%) tumors in FGS group and fifteen (42.9%) tumors in non-FGS group were identified as deep-seated tumors, respectively. No obvious difference was observed in the location characteristics of the two groups. In terms of histology, according to the WHO 2021 classification of gliomas, all lesions included in the study were grade 3 or 4 gliomas (HGGs, 27 (84.4%) grade 4 in FGS vs. 28 (80.0%) grade 4 in non-FGS). Glioblastoma accounts for the highest proportion in the two groups, followed by H3 K27-altered diffuse midline glioma (DMG), astrocytoma, and oligodendroglioma. We also compared the MGMT promoter methylation, IDH, and H3K27M mutation status between the two groups and found that, apart from a trend towards unmethylated lesions being more common in the FGS group (without statistical significance), there were no differences in the glioma molecular profiling between the two groups (Table 1). Overall, there were no significant differences in demographic and clinical baseline characteristics between FGS and non-FGS.

**Table 1:** Summary of demographics, lesion location, and pathology

Characteristic	FGS (*n* = 32)	Non-FGS (*n* = 35)	*p* value
Age (mean (SD)), years	41.9 (14.6)	46.2 (15.8)	0.257
Sex (%)			0.628
Male	17 (53.1)	16 (45.7)	
Female	15 (46.9)	19 (54.3)	
Side (%)			0.634
Middle	5 (15.6)	3 (8.6)	
Left	17 (53.1)	16 (45.7)	
Right	9 (28.1)	15 (42.9)	
Bilateral	1 (3.1)	1 (2.9)	
Functional location grade (%)			1.000
II	10 (31.2)	12 (34.3)	
III	22 (68.8)	23 (65.7)	
Deep-seated (%)	12 (37.5)	15 (42.9)	0.804
Preoperative tumor volume (median [IQR]), cm^3^	20.3 [11.2, 36.9]	23.7 [12.9, 37.7]	0.787
WHO CNS5 classification (%)			0.653
Astrocytoma, IDH-mutant, CNS WHO grade 3/4	2 (6.2)	3 (8.6)	
Oligodendroglioma, IDH-mutant, 1p/19q-codeleted, CNS WHO grade 3	2 (6.2)	2 (5.7)	
Diffuse pediatric-type high-grade glioma, H3-wildtype and IDH-wildtype, CNS WHO grade 4	0	1 (2.9)	
Diffuse midline glioma, H3 K27-altered, CNS WHO grade 4	11 (34.4)	7 (20.0)	
Glioblastoma, IDH-wildtype, CNS WHO grade 4	17 (53.1)	22 (62.9)	
WHO CNS5 grade 4 (%)	27 (84.4)	28 (80.0)	0.755
MGMT promoter status (%)			0.088
Methylated	12 (37.5)	21 (60.0)	
Unmethylated	20 (62.5)	14 (40.0)	
IDH status (%)			0.736
Wildtype	28 (87.5)	29 (82.9)	
Mutant	4 (12.5)	6 (17.1)	
H3K27M status (%)			0.270
Wildtype	21 (65.6)	28 (80.0)	
Mutant	11 (34.4)	7 (20.0)	

**Fig. 3:**
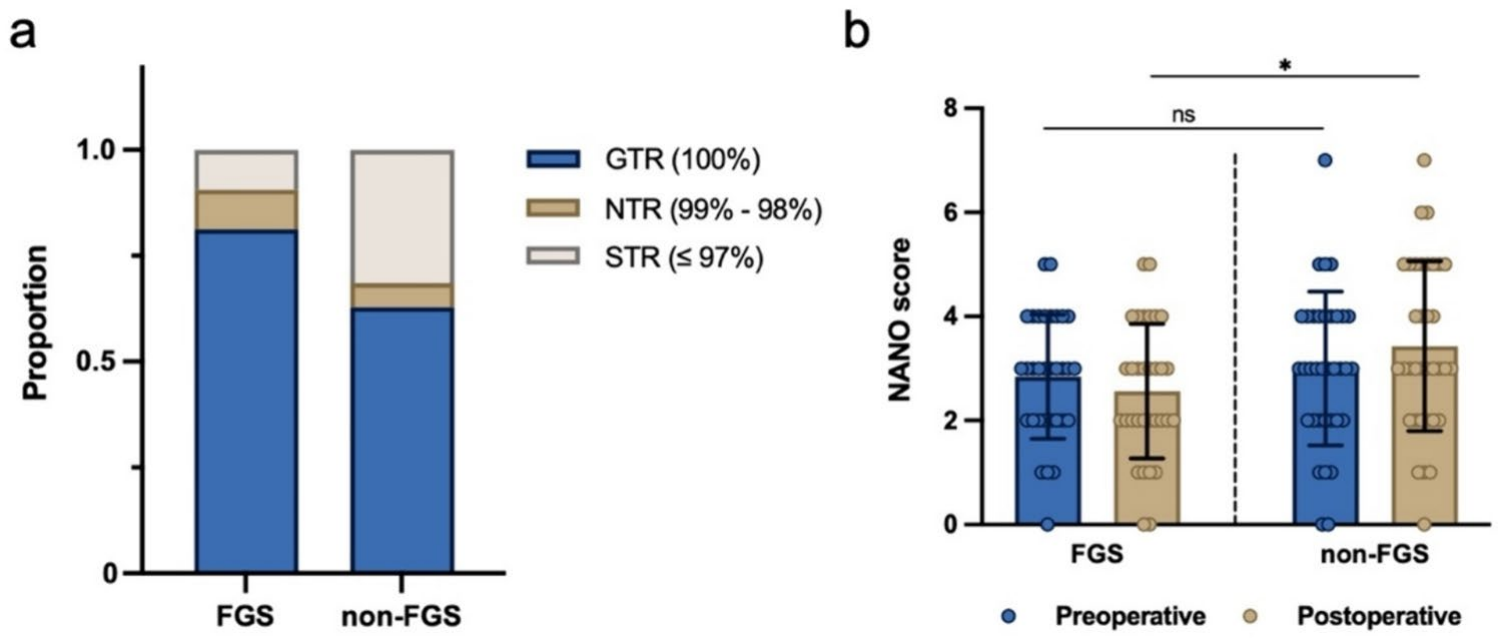
**a** Stacked bar graph shows proportion of stratified EOR between the FGS group and non-FGS group. **b** The comparison of preoperative and 3-month postoperative NANO score regarding the usage of fluorescence sodium. *ns* no significance. *p < 0.05

Usage of sodium fluorescein during surgery exhibited increased GTR and NTR rate over white-light surgery (Fig. 3a). GTR was achieved in 27 tumors (84.4%) in the FGS group, which was significantly higher than 21 tumors (60.0%) in the non-FGS group (OR 3.60, 95% CI 1.18–10.28, *p* < 0.05) (Table 2). No significant difference was noted in the number of cases achieving NTR between the two groups. Upon combining GTR and NTR, the FGS group showed a notably higher frequency of cases achieving ≥ 98% EOR compared to the non-FGS group (93.8% vs. 65.7%, OR 7.83, 95% CI 1.86–36.85, *p* < 0.01). Within tumors impacting eloquent regions, the FGS group demonstrated a markedly higher EOR of achieving GTR or NTR, with 20 tumors (87.0%) compared to only 12 tumors (52.2%) in the non-FGS group (OR 6.11, 95% CI 1.50–22.78, *p* < 0.05). Similar result was shown on EOR of deep-seated tumors. The rate of GTR + NTR in the study group was significantly higher than the control group (91.7% vs. 53.3%, OR 9.62, 95% CI 1.05–116.50, *p* < 0.05) (Table 2).

**Table 2:** Analysis of EOR and survival status

	FGS	non-FGS	OR/HR (95% CI)	*p* value
EOR of all tumors				
GTR (100%)	27 (84.4%)	21 (60.0%)	3.60 (1.18–10.28)	0.033
NTR (99 to 98%)	3 (9.4%)	2 (5.7%)	1.71 (0.33–10.06)	0.664
GTR + NTR (≥ 98%)	30 (93.8%)	23 (65.7%)	7.83 (1.86–36.85)	0.006
STR (≤ 97%)	2 (6.3%)	12 (34.3%)	0.13 (0.03–0.54)	0.006
EOR of eloquent tumors (grade III)				
GTR + NTR (≥ 98%)	20 (87.0%)	12 (52.2%)	6.11 (1.50–22.78)	0.023
EOR of deep-seated tumors				
GTR + NTR (≥ 98%)	11 (91.7%)	8 (53.3%)	9.62 (1.05–116.50)	0.043
Median survival (months)				
OS	18.2	14.0	0.63 (0.36–1.11)	0.112
PFS	11.2	7.7	0.59 (0.35–0.99)	0.036

Patients exposed to sodium fluorescein experienced no harmful adverse events intraoperatively and postoperatively, except for transient yellowish urine. No serious postoperative complications (grade III and IV) were observed in either group of patients. Each group had one case of postoperative hematoma in the surgical area. The hematoma in the FGS group was gradually absorbed following close monitoring and intracranial pressure management. The other one in the non-FGS group underwent hematoma evacuation surgery with a favorable outcome. In the non-FGS group, a case of radiation necrosis and marked cerebral edema resulted in neurologic impairment but without lethal outcomes.

**Fig. 4:**
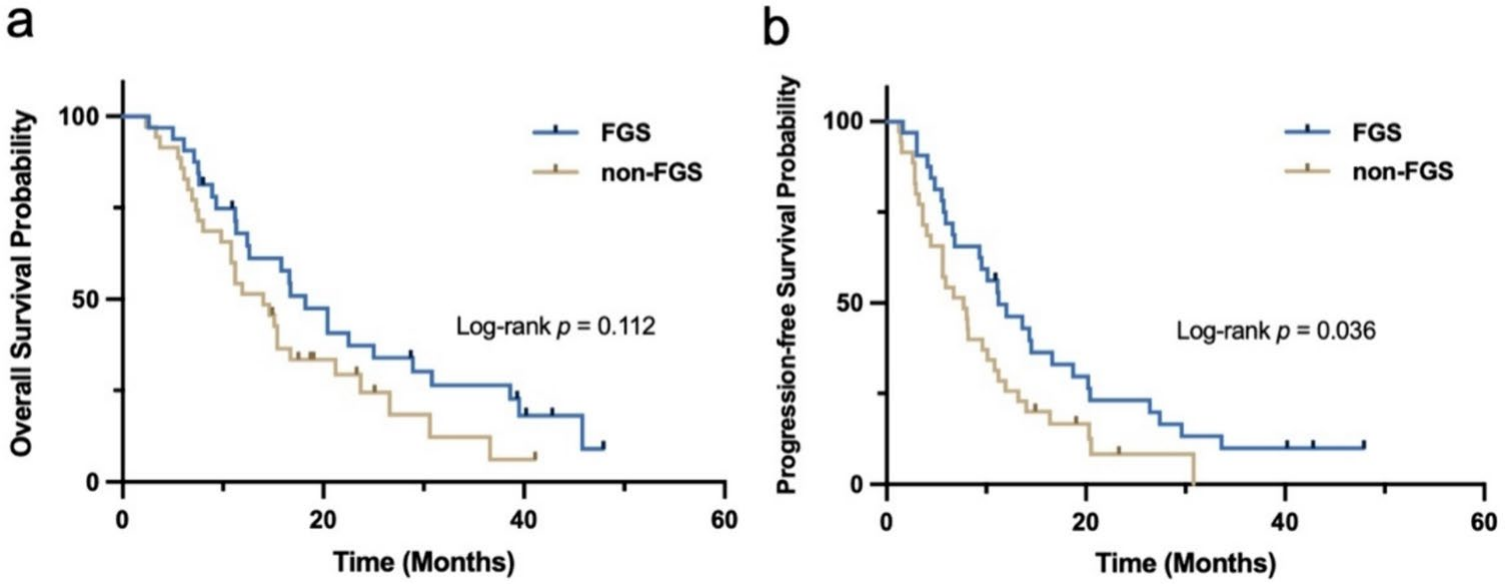
Kaplan-Meier curve of OS (**a**) and PFS (**b**)

Preoperatively, both groups showed similar NANO scores (2.8 ± 1.2 vs. 3.0 ± 1.5, *p* = 0.637); nonetheless, at the 3-month follow-up, the FGS group displayed notably lower scores than the non-FGS group (2.6 ± 1.3 vs. 3.4 ± 1.6, *p* < 0.05), suggesting a superior safety profile for FGS (Fig. 3b). The median OS in the FGS group was 18.2 months, which, though 4.2 months longer than that in the non-FGS group (14.0 months), showed no significant difference between the two (Fig. 4a; Table 2). However, PFS for the FGS group was significantly longer than the non-FGS group (11.2 months vs. 7.7 months, HR 0.59, 95% CI 0.35–0.99, *p* < 0.05) (Fig. 4b; Table 2).

The cohort was then divided into two groups: adult-type tumors (astrocytoma, oligodendroglioma, and GBM) and pediatric-type tumors (DMG and pHGG), based on the WHO CNS5 classification, and a stratified analysis was performed. A higher proportion of cases in the FGS group achieved an EOR rate exceeding 98% compared to the non-FGS group, particularly in cases involving grade III eloquent areas, for both adult and pediatric tumors. However, in cases of deep-seated tumors, only the FGS group for pediatric tumors exhibited such an advantage in EOR (Supplementary Table 1). In terms of neurologic assessment, the 3-month postoperative NANO scores were significantly higher in the FGS group for pediatric tumors compared to the non-FGS group (2.9 ± 0.9 vs. 4.4 ± 1.7, *p* < 0.05), but no significant difference was observed in adult tumors. There was no notable distinction in OS between the FGS and non-FGS groups for both tumor types. However, in pediatric tumors, the FGS group exhibited a significantly longer PFS than the non-FGS group (9.3 months vs. 4.4 months, *p* < 0.05). A similar pattern was observed in adult tumors, but the results were not statistically significant (Supplementary figure).

## Discussion

In the present study, we retrospectively analyzed data from our institution on newly diagnosed high-grade gliomas located in eloquent and deep-seated areas, treated with sodium fluorescein-assisted surgery over the past four years. The study aimed to compare the extent of resection, neurologic function changes, and survival outcomes between fluorescence-guided surgery and conventional white-light surgery.

For high-grade gliomas, the current standard of care remains maximal safe resection, with the critical focus on striking a balance between the extent of resection and the preservation of neurologic function to truly benefit patients. As a result, an increasing number of tools are being employed to enable more precise identification of tumor boundaries and achieve broader resection margins, including intraoperative navigation, iMRI, ultrasonography, fluorophores, and real-time mutational analyses. 5-ALA and sodium fluorescein are the most used fluorescent agents intraoperatively. Their mechanisms of action differ, with 5-ALA identifying tumor cells by distinct metabolic products in normal and tumor cells, while sodium fluorescein, requiring no metabolic process, accumulates in the intercellular spaces at sites of disrupted BBB. The intraoperative intensity of fluorescein is directly proportional to the invasiveness of the tumor, in line with its mechanism of action. Indeed, helpful intraoperative fluorescence was identified by surgeons in most tumors of neuroepithelial tissue (HGGs, ependymoma, and glioneuronal tumor), metastases, and primary CNS lymphomas, but not in LGGs, meningiomas, and hemangioblastomas, reported by the FLUOCERTUM study (Falco et al. 2019). In addition, research on pediatric LGGs-FGS demonstrated that 86% of the lesions showed fluorescence uptake, with 74% of them being helpful for resection (de Laurentis et al. 2022). In line with the previous results, satisfactory fluorescence of tumor tissues under 560 nm filter was visualized in all 32 cases of HGGs undergoing fluorescence-guided resection included in the present study. The collective evidence from several studies indicates a sensitivity of 82–94% and a specificity of 90–91% in the identification of HGGs using fluorescence (Senders et al. 2017). A meta-analysis of 12 studies revealed a consistent pooled sensitivity of 84% and specificity of 91% in detecting tumor tissue with fluorescence (Katsevman et al. 2020). These findings suggest that fluorescein serves as a dye that can visualize HGGs, effectively marking the tumors.

Growing evidence from studies on the EOR between FGS and white-light surgery consistently indicates that fluorescence guidance allows for the removal of a greater volume of tumor tissue with minimal postoperative complications (Hong et al. 2019; Katsevman et al. 2020; Xue et al. 2021; Schebesch et al. 2022; Xi et al. 2023). Hansen et al. (2019) reported a median reduction of tumor volume at 97.4% (IQR 90.8–100%) with fluorescein. Schebesch et al. (2022) further extended the EOR to median 100.0% (range 61.6–100.0%). However, there is considerable variation among studies in terms of the GTR rates. GTR of 62% in the fluorescence group was achieved when compared to 5-ALA in Hansen’s study, which retrospectively analyzed 209 HGGs (Hansen et al. 2019). Xi et al. (2023) compared the GTR rates between 61 cases of FGS and 51 cases of non-FGS, which were 45.9% and 19.6%, respectively. The authors attributed the lower GTR rates in the above two studies than others to the utilization of the RANO criteria to define EOR (Wen et al. 2023). A meta-analysis by Smith et al. (2021) enrolled 21 studies regarding intraoperative fluorescence, including 11 single arm studies without control group and 10 high quality case-controlled studies, which was integrated for the analysis. The pooled GTR rates in the FGS group of 449 participants and the non-FGS group of 379 participants were 80.2% and 50.7%, respectively, as revealed by the results. Subsequent small-scale studies have indicated similar findings, with GTR of 82% and 85% (Xue et al. 2021; Falco et al. 2022). Our data showed that GTR of FGS group and non-FGS group were 84.4% and 60.0%, respectively, aligning closely with previous studies. A well-established research finding indicates a strong correlation between excising over 98% of tumors and favorable prognosis (Lacroix et al. 2001). In light of this, we further analyzed the GTR + NTR rates (EOR ≥ 98%) in the two groups, which were 93.8% and 65.7% (OR 7.83, 95% CI 1.86–36.85, *p* < 0.01), respectively, both higher than previous studies (Katsevman et al. 2020).

Previous research on fluorescence-guided resection of HGGs in different areas only covered the scope of the cerebral lobes, lacking specific studies on tumors located in the eloquent areas and deep structures of the brain (Hong et al. 2019; Katsevman et al. 2020; Zeppa et al. 2022). Thus, it remains unclear whether patients would benefit from the use of fluorescence-guided resection for these challenging tumors. Xi et al. (2023) revealed that tumors in the temporal and occipital lobes had a larger EOR under fluorescence guidance, while no significant difference was observed within the frontal lobe, parietal lobe, and deep supratentorial region. The authors noted that although their research analyzed tumors in different brain regions, it did not specifically differentiate between eloquent and non-eloquent areas. Although another prospective study identified the eloquence grades of tumors, analysis of the outcomes following fluorescence-guided resection at different tumor grades was not conducted (Acerbi et al. 2018). Moreover, some studies have ruled out infratentorial and deep-seated lesions (Acerbi et al. 2013, 2018). Therefore, in this study, we screened grade II and grade III tumors based on the Lacroix functional location grading (Lacroix et al. 2001), and incorporated both supratentorial and infratentorial HGGs. Our data indicated that the GTR + NTR rate of eloquent tumors (grade III) was higher in the FGS group than the non-FGS group, as well as in deep-seated tumors. Theoretically, IONM could restrict the EOR under FGS. However, based on our practical results, the simultaneous application of these intraoperative techniques helps to clarify safe resection margins and maximize the extent of resection. Given our experience with IONM and surgery, along with the fact that our study involved tumors located near eloquent areas, rather than exclusively focusing on highly eloquent (motor and language) areas, the results of the present study support our findings on the extent of resection. Acerbi et al. (2018) utilized identical functional classification of HGGs as our study, applied IONM in cases classified as grade II and III. The results revealed that out of 31 tumors classified as grade II or III, 28 (90.3%) achieved an EOR > 98%. Of these, 20 were classified as grade III, with 17 (85%) achieving an EOR > 98%. Another study conducted by Falco et al. (2022) demonstrated that out of 28 patients with eloquent GBM resection assisted by sodium fluorescein and IONM, 25 cases (89.3%) achieved an EOR rate of over 98%. These findings were consistent with ours (93.8% for grade II and III, 87% for grade III).

We further investigated whether the expansion of EOR could result in a prolonged survival. The median OS showed no significant difference between the FGS group and the non-FGS group (18.2 months vs. 14.0 months, HR 0.63, 95% CI 0.36–1.11, *p* = 0.112), although 4.2 months longer of FGS than non-FGS. The limited sample size in the study could be a contributing factor, along with the fact that, despite the lack of statistical difference between the two groups, the FGS group included a higher proportion of patients with unmethylated MGMT promoter, a known pivotal determinant of OS (Table 1). A longer PFS observed in the study group (11.2 months vs. 7.7 months, HR 0.59, 95% CI 0.35–0.99, *p* < 0.05) is attributed to its increased proportion of cases with the EOR ≥ 98%. Our findings are in line with previous studies. Hansen et al. (2019) reported the fluorescence group achieved a 19.7-month OS, although without control group. Another study by Schebesch and collogues revealed the median OS between the fluorescence group and the white light group were 16.7 months and 15.5 months, and the median PFS were 8.12 months and 6.94 months, respectively (Schebesch et al. 2022). Katsevman et al. (2020) also reported that patients with GBM who underwent surgery with sodium fluorescein received an 18-week longer OS than those without fluorescence (mean 78 weeks vs. mean 60 weeks). While our EOR showed a slight improvement over other studies, there was no significant increase in survival time compared to them. This discrepancy may be due to the incorporation of a subset of DMG patients with unfavorable prognoses in our study.

To investigate the safety of fluorescein-guided surgery, we also conducted the neurologic function estimation using the NANO scale system. The author of RANO 2.0 indicated that most widely used performance status scores, Karnofsky performance status (KPS), cannot assess the neurologic function of patients with gliomas accurately (Youssef and Wen 2024). The NANO scale is a well-designed scoring system which is easy to perform at bedside. The postoperative NANO score of the FGS group was significantly lower than the non-FGS group (2.6 ± 1.3 vs. 3.4 ± 1.6, *p* < 0.05), suggesting that the EOR improvement with sodium fluorescein did not result in safety risks, as indicated by the maintenance of neurologic function in patients receiving FGS, which was even superior to the control group. This favorable outcome is linked to the ability of fluorescence-guided techniques to differentiate tumor boundaries more accurately from adjacent brain tissue. The preservation of neurological function also relies on the integration of multiple tools, including DTI, fMRI, and IONM.

Deep-seated tumors tend to be located near eloquent areas. Indeed, one third of the lesions were both grade III and deep-seated tumors in our cohort. To assess the efficacy of fluorescein in tackling these challenging tumors, we included pediatric tumors (mostly DMGs) in the study to provide a greater representation of deep-seated tumors. We conducted a stratified analysis for different tumor types. The results revealed that, for eloquent tumors, FGS achieved a higher EOR compared to the non-FGS group, in both adult and pediatric tumors. However, for deep-seated tumors, only pediatric cases with FGS exhibited an advantage, resulting in a longer PFS. This may be attributed to the fact that DMGs predominantly affect midline structures, particularly the brainstem, enabling fluorescein to guide surgeons in accurately identifying tumor tissue within a very limited space.

**Fig. 5:**
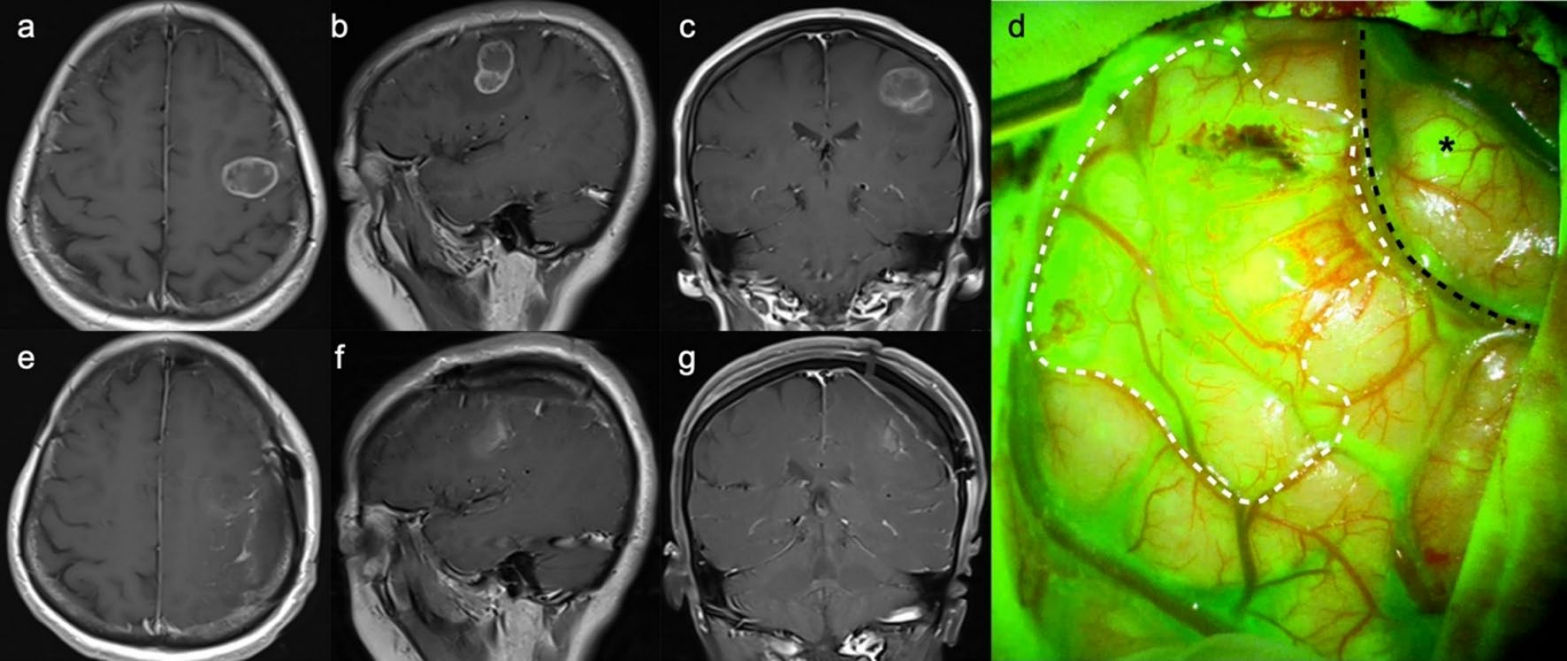
MR images and intraoperative fluorescence of a 47-year-old female with eloquent glioblastoma. **a**–**c** Preoperative CE T1 WI images reveal that the lesion was located in the left posterior inferior frontal gyrus and anterior to the precentral gyrus, accompanied by a septated cyst and multiple patchy enhancement. **e**–**g** Postoperative CE T1 WI indicates a complete resection of the tumor through a frontoparietal craniotomy. **d** The tumor displayed bright fluorescence with well-defined borders against the surrounding brain parenchyma. A small satellite lesion (asterisk), undetected on MRI, was noted within the precentral gyrus. The tumor’s boundary on the surface of brain is delineated with a white dashed line, and the central sulcus is outlined by a black dashed line

Fluorescein is considered to label a similar extent to that of contrast-enhanced T1-weighted MRI. However, studies on fluorescence-guided aggressive resection of glioblastoma have shown that fluorescence-positive areas extend beyond the contrast-enhancing regions. This discrepancy may be attributed to differences in vascular permeability and tissue penetration between fluorescein and gadolinium. One case in our study shown a transsulcal satellite lesion located remotely from the core lesion, which could not be observed in CE T1 WI MRI (Fig. 5). This finding suggests that fluorescein may more readily penetrate the brain parenchyma in the early stages of BBB disruption. While the exact reasons remain unclear, this finding amplifies the potential value of fluorescein in HGGs surgery.

The study is constrained by its limited number of cases, which hinders its ability to provide a robust understanding of long-term overall survival outcomes. Additionally, the incorporation of various WHO CNS grade 3 and 4 tumors introduced heterogeneity into the study. Moreover, we did not conduct awake craniotomy in patients with tumors in language eloquent areas. Instead, we utilized multimodal techniques, such as integrating preoperative BOLD-fMRI and language tracts data into neuronavigation for surgery design, as well as DCS/DsCS and IONM for achieving maximal safe resection. Within our study cohort, 6 cases (9.0%) had lesions near the traditional language area, with 3 cases presenting with dysarthria preoperatively, and only 1 case showing mild worsening in the language domain of the NANO scale postoperatively. Nevertheless, it would be beneficial to evaluate the potential value of FGS in the resection of tumors in highly eloquent (motor and language) area under awake craniotomy.

## Conclusion

Compared to conventional surgery under white-light microscope, using sodium fluorescein-guidance surgery for high-grade gliomas located in eloquent and deep-seated areas of the brain allows for a larger extent of resection without compromising neurologic function, thus potentially prolonging survival time and facilitating precise treatment for these challenging tumors.

### Supplementary Information

Below is the link to the electronic supplementary material.

Supplementary file1 (JPG 3485 KB)

Supplementary file2 (DOCX 18 KB)

Supplementary file3 (XLSX 19 KB)

## Data Availability

The datasets generated during the current study is available from the corresponding author. No datasets were generated or analysed during the current study.
