# Supplementary material for: Fluorescein-guided surgery in high-grade gliomas: focusing on the eloquent and deep-seated areas
Source: J Cancer Res Clin Oncol. 2024 May 25;150(5):274. doi: 10.1007/s00432-024-05796-1 (PMC11127876; doi:10.1007/s00432-024-05796-1)
Supplement: Supplementary file 2 — Supplementary file2 (DOCX 18 KB) [file 432_2024_5796_MOESM2_ESM.docx]

|  | **Adult tumors** | | | |  | **Pediatric tumors** | | | | |
| --- | --- | --- | --- | --- | --- | --- | --- | --- | --- | --- |
|  | FGS | non-FGS | OR/HR (95% CI) | *p* value |  | FGS | non-FGS | OR/HR (95% CI) | *p* value |  |
| EOR of all tumors |  |  |  |  |  |  |  |  |  |  |
| GTR (100%) | 19 (86.4%) | 18 (66.7%) | 3.17 (0.77-11.96) | 0.182 |  | 9 (81.8%) | 3 (37.5%) | 7.50 (1.05-47.34) | 0.074 |  |
| NTR (99% - 98%) | 2 (9.1%) | 1 (3.7%) | 2.60 (0.28-38.92) | 0.581 |  | 1 (9.1%) | 0 | - | >0.999 |  |
| GTR+NTR (≥ 98%) | 21 (95.5%) | 19 (70.4%) | 8.84 (1.35-102.1) | 0.031 |  | 10 (90.9%) | 3 (37.5%) | 16.67 (1.57-210.60) | 0.041 |  |
| STR (≤ 97%) | 1 (4.5%) | 8 (29.6%) | 0.11 (0.01-0.74) | 0.031 |  | 1 (9.1%) | 5 (62.5%) | 0.06 (0.01-0.64) | 0.041 |  |
| EOR of eloquent tumors (grade III) |  |  |  |  |  |  |  |  |  |  |
| GTR+NTR (≥ 98%) | 13 (92.9%) | 8 (53.3%) | 8.67 (1.29-136.10) | 0.017 |  | 7 (87.5%) | 3 (60%) | 11.67 (1.00-152.80) | 0.039 |  |
| EOR of deep-seated tumors |  |  |  |  |  |  |  |  |  |  |
| GTR+NTR (≥ 98%) | 5 (100%) | 6 (54.5%) | - | 0.119 |  | 6 (85.7%) | 1 (25%) | 18 (1.06-261.20) | 0.044 |  |
| Median survival (months) |  |  |  |  |  |  |  |  |  |  |
| OS | 22.5 | 15.4 | 1.46 (0.76-2.80) | 0.133 |  | 15.8 | 8.9 | 1.79 (0.66-4.79) | 0.136 |  |
| PFS | 14.3 | 8.0 | 1.9 (0.97-3.29) | 0.070 |  | 9.3 | 4.4 | 0.24 (0.07-0.78) | 0.018 |  |

**Supplementary table 1. Stratified analysis of EOR and survival status in adult and pediatric tumors.**
